# Self-expansion is positively associated with Fitbit-measured daily steps across 4-weeks

**DOI:** 10.1038/s41598-022-24576-w

**Published:** 2022-11-29

**Authors:** Xiaomeng Xu, Samantha Tupy, Julia L. Sharp, Ashley L. Miller, Danielle Correll, Claudio R. Nigg

**Affiliations:** 1 Department of Psychology, Idaho State University, Pocatello, ID USA; 2 Office of Public Health Studies, University of Hawai’i at Manoa, Honolulu, HI USA; 3 Department of Statistics, Colorado State University, Fort Collins, CO USA; 4 Present Address: Department of Veteran Affairs, Fargo, ND USA; 5 Present Address: Department of Psychology, UCLA, Los Angeles, CA USA; 6 Present Address: Department of Psychology, University of Colorado, Colorado Springs, CO USA; 7 Present Address: Institute of Sports Science, University of Bern, Bern, Switzerland

**Keywords:** Psychology, Human behaviour

## Abstract

The growth of the self-concept through increasing perspectives, identities, resources, and efficacy is known as self-expansion and typically involves novelty, challenge, interest, and/or excitement. Self-expansion is positively associated with health factors including self-reported physical activity (PA). This study is the first to investigate self-expansion and daily PA, and with a PA monitor. Fifty community participants completed baseline questionnaires, wore a Fitbit One and completed daily self-report questionnaires for 28 days, and completed follow-up questionnaires. Daily surveys included questions about both general and PA-specific self-expansion. Across the 4 weeks, steps taken was positively correlated with both general (all maximum likelihood *r* = 0.17) and PA-specific self-expansion (maximum likelihood *rs* of 0.15 and 0.16), and PA-specific self-expansion was positively correlated (maximum likelihood *rs* of 0.38 and 0.50) with aerobic activity. Future research should investigate this relationship in a larger more diverse sample and test whether PA-specific self-expansion can be utilized as an acceptable, feasible, and effective intervention to increase daily steps and other forms of PA.

## Introduction

Physical activity (PA) provides many health benefits including protection from chronic illnesses (e.g., cardiovascular disease, cancer), mental health conditions (e.g., depression), and premature death^1^. Walking is one of the most common forms of PA and daily steps are positively associated with health, with 10,000 steps per day as a common recommendation^2–6^. Adults typically do not meet this recommendation however^2^, thus research on factors that positively affect daily steps is important.

One such factor is self-expansion. The self-expansion model^7^ states that people are intrinsically motivated to enhance their abilities to achieve goals by increasing the number and/or positive content of perspectives, identities, and resources; that is, people pursue activities that increase the positive content of their self-concept. While the self-expansion model was first developed to better understand close relationships, more recently self-expansion theory and research has encompassed experiences at the individual level. Self-expanding activities are characterized by novelty, interest, challenge, and or/excitement with examples including engaging in hobbies, learning new skills, establishing new social relationships, and engaging in intellectual and spiritual experiences.

Self-expansion is rooted in approach motivation^8^ and positively influences perceptions of and motivation to engage in opportunities for self-expansion as well as exerted effort^8,9^. Having a positively-valanced and robust self-concept is also protective in terms of psychological distress, with recent research showing that self-expansion is negatively associated with depression symptoms across cross-sectional, daily, and longitudinal methodologies^10^. This protective role in the context of mental health provides another avenue through which self-expansion may be helpful when individuals are contemplating taking on a potentially challenging task such as changing one or more behaviors. Given the increase to positive self-concept content and typical factors (e.g., novelty, excitement) of self-expanding activities, it is unsurprising that self-expansion is associated with positive affect^11^. However, it is important to note that self-expansion is not simply positive affect, and engagement in self-expanding experiences leads to better outcomes than engagement in just positive experiences^12^. Finally, because self-expansion also increases positive content to an individual’s self-concept (e.g., feeling like one is growing as a person because of increasing perspectives, identities, skills etc.), it leads to greater self-efficacy^13^ which has important practical implications for behavior and behavior change. Having greater confidence in oneself to be able to perform a specific behavior is associated with positive changes in intention and that behavior^14^. That is, greater self-efficacy makes it more likely that a person feels ready to take on a potentially challenging task such as behavior change, and is more likely to persist in their attempts even when setbacks may occur. Because increasing positive content to the self-concept is associated with many factors that positively influence behavior change (approach motivation, effort, positive mental health, self-efficacy, positive affect) self-expansion may be especially useful to examine in the context of health research.

Research on self-expansion shows that it is associated with a number of health variables including health behaviors. For example, self-expansion is positively associated with cigarette abstinence, and individuals who report experiencing a greater number of self-expanding experiences (e.g., learning a new skill, joining a club, making a new friend, positive change at work, starting a new type of recreation or exercise) are more likely to successfully quit smoking^15^. FMRI studies have also shown attenuation of neural cigarette cue-reactivity in overnight abstinent smokers when those individuals experimentally engaged with self-expanding stimuli compared to pleasant control stimuli^16,17^. Self-expansion has also been proposed as one factor to consider in exercise prescriptions^18^. In one study^19^, researchers assessed self-reported levels of self-expansion with a Likert-type scale as part of a 12-week behavioral weight loss intervention of community adults whose Body Mass Indexes were in overweight or obesity ranges. Self-expansion was significantly associated with weight loss, likelihood of achieving clinically significant weight loss (≥ 5% of body weight), and self-reported PA during the 12-weeks of the intervention.

To date, no study has examined the relationship between self-expansion and *objectively-measured* PA of any kind, nor this association at the daily level (in^19^, participants reported on self-expansion for the prior 12 weeks). The current study sought to elucidate the association between self-expansion and objectively measured daily steps. Specifically, we hypothesized that in line with previous research on self-expansion and behavioral health factors, self-expansion would be positively associated with objectively measured daily steps. The study is also the first to investigate domain-specificity for self-expansion by examining PA-specific self-expansion (e.g., novelty, excitement related to PA) in addition to general self-expansion (e.g., novelty, excitement in daily life). Understanding these hypothesized existing naturalistic associations between self-expansion and objectively-measured steps is a necessity before establishing a future intervention that would target steps or other PA through increasing self-expansion.

## Method

### Design

The study was a longitudinal design with daily assessments over four weeks, with the outcome of interest being the association between general and PA-specific self-expansion and an objective PA indicator (steps).

### Participants

Utilizing the small effect size from^12^, which at the time of this project was the only daily self-expansion study, focusing on couples’ relationships, a within-subjects repeated measure design over 28 days, and 0.05 significance level, 30 participants were needed to achieve 80% power to detect an association across time (G*Power^20^). We chose a sample size of 50 to mitigate potential adherence/retention issues. The Human Subjects Committee of Idaho State University approved the study, the research was performed in accordance with the Declaration of Helsinki, and participants provided informed consent.

Fifty adults were recruited from the Southeast Idaho community using flyers, email listservs, and word of mouth. Participants were 39.96 years of age on average (*SD* = 12.14, range = 21–73), with 72% of participants identifying as women, 26% men, and 2% gender fluid. The majority (88%) identified as non-Hispanic White, reflecting the local population. The rest identified as Asian-American (4%), Hispanic (4%), African-American (2%), and American / Alaska Native (2%).

### Measures

Objective daily steps were measured with the Fitbit One. Fitbit is a popular commercial PA monitor and offers several models utilized in research. Studies have demonstrated that the Fitbit One is a device that is typically worn at the hip, clipped to clothing, and a valid and reliable device for measuring daily number of steps^21,22^, comparable to an Actigraph^23^. The monitor automatically uploads data to the Fitbit website via the Internet, so wearers do not need to record or enter any data. Wearers are able to access their data from the device or the Fitbit website. Because this was a naturalistic study and not an intervention (participants were asked to maintain their current PA levels and not change their habits), no activity goals were set. We set a steps cutoff such that a day would be invalid if total steps for that day was below the cutoff. Steps cutoffs for valid days from 300–1,500 steps have been used in prior research^24–26^. We opted for the most stringent cutoff, treating anything under 1,500 steps as non-wear and invalid data.

General and PA-specific self-expansion were measured with online surveys prior to the lab visits. Both were assessed with modified versions of the Self Expansion Questionnaire (SEQ;^27^), which is the first and most commonly used measure of self-expansion (e.g.,^8^). The SEQ has 14-items with a 1 (not very much) to 7 (very much) response scale. Item scores are summed to create a total score. Because the SEQ focuses on self-expansion within romantic relationships with all questions about the partner or relationship, we modified the measure so all items were appropriate for our sample and design. This type of modification for the SEQ is standard in the literature when non-relational self-expansion is examined (e.g.,^9^). For example, the item “Do you often learn new things about your partner” was modified to “Do you often learn new things” for the general self-expansion measure and “Do you often learn new things about physical activity” for the PA-specific self-expansion measure. Due to an error, for the PA-specific self-expansion questionnaire, two items were omitted from the online survey and thus the measure had 12 items. We cannot be certain of the effect of the omission, however previous self-expansion research has successfully utilized shorter modified versions of the general self-expansion questionnaire (e.g.,^28^ used a five-item measure in multiple studies). Further, while we examined baseline and follow-up PA self-expansion, our main analyses focused on specific self-expansion items that were asked each day rather than the full measures which were only administered during baseline and follow-up. In the current study, the general self-expansion questionnaire had high reliability (Cronbach’s α = 0.95 at baseline and 0.97 at follow-up), as did the PA-specific self-expansion questionnaire (Cronbach’s α = 0.98 at baseline and 0.98 at follow-up).

Daily surveys asked participants to report “How much TODAY” they experienced general and PA-specific self-expansion (responses options were Not at All, A Little, Somewhat, A Good Deal, Very Much, or Not Applicable). To reduce participant burden, only 3 questions (based on items used in^19^) were asked about daily general self-expansion (novelty, excitement, growth). Reduction of the number of items to assess for self-expansion is common in the literature (e.g.,^28^). Two questions about PA-specific self-expansion (excitement, novelty) were asked daily. If participants indicated that they engaged in aerobic activity that day, they were also asked about excitement and novelty of those aerobic activities for that day. The same five-point scale was used for all daily items for consistency and to enhance readability on mobile screens.

### Procedure

The protocol for this study including strategies for the high adherence and retention rates have been published previously^29^. Briefly, the study consisted of two in-person laboratory visits (pre and post study) and four weeks of daily online surveys and Fitbit wear. Inclusion criteria for participants were being at least 18 years of age, English speaking, absence of medical issues that would prevent walking, and willingness to complete study tasks including wearing the Fitbit and completing a survey daily for the four weeks.

During the first lab session, study staff gave participants the Fitbit and oriented them to the device (e.g., how to wear, charging and syncing, that it wasn’t waterproof). Participants were reminded of their research start date, that they were not being asked to change their behavior as this was not an intervention study, and that they would be receiving an automated daily email which would include a time-sensitive survey link (they were to complete the survey immediately before going to bed). Participants were also reminded in these daily emails to wear their devices each day and sync and charge their devices regularly.

At the end of the four weeks, participants completed online follow-up questionnaires and returned to the lab where they were thanked and provided with compensation (prorated payment of up to $50 and the Fitbit device based on level of completion). Participants’ steps data for the four weeks were also downloaded from the Fitbit website and added to our de-identified data files.

### Data analyses

Matched pairs t-tests were used to compare baseline and follow-up general and PA-specific self-expansion and steps. Simple linear regression analysis was used to examine the relationship between the average daily steps and each of baseline general and PA-specific self-expansion. For the primary aim, daily correlations between daily steps and self-expansion measures were computed using SAS PROC CORR (SAS v9.4). SAS PROC MIXED was used to obtain maximum likelihood estimates of the correlation coefficient^30,31^ between each self-expansion measure and number of steps. This method estimates the overall correlation coefficient between two variables in two parts with and without the subject effect. Days nested within subjects was set as the repeated factor with an autoregressive variance structure which allows for the correlation of observations on the same subject to decrease over time^30,31^. The use of the mixed effects model also allowed for inclusion of individuals with missing observations. SAS PROC MIXED was also used to examine the association between each of the self-expansion measures and the number of steps (response) over time and if the response varied by self-expansion and day (interaction). A random intercept and slope for day for each participant was included in the model. An autoregressive variance structure was used to account for repeated measures on the same subjects over time. Kenward-Roger approach was used to estimate degrees of freedom^32^. PROC PLM was used to estimate slopes between self-expansion measures and average number of steps for each day when the interaction between self-expansion and day was significant in the model. A significance level of 0.05 was used for all tests of significance.

## Results

All 50 participants completed the study and both lab visits. Out of the 28 daily surveys, participants completed an average of 27.12 (*SD* = 3.27). Forty-nine participants (98%) completed at least 25 of the 28 daily questionnaires. The remaining participant (a 28-year old non-Hispanic White man) only completed five questionnaires, which was below our a priori minimum of at least two weeks (50%) of data, and was removed from the dataset for subsequent analyses. This participant did not provide feedback on their low compliance. Synched Fitbit data existed for participants on average for 27.32 days (*SD* = 1.25, range 23–28). Using the cutoff established for the total number of daily steps (at least 1500 steps), there were 30 (2.19%) invalid days (pairwise deleted in correlation analyses), and a final total of 1,342 valid days in our dataset. Forty-six participants had both baseline and 4-week follow-up steps logged.

Participants’ baseline general self-expansion was moderately high on average (*M* = 73.96, *SD* = 15.62 out of 98), as was their PA-specific self-expansion (*M* = 62.51, *SD* = 18.50 out of 84). There was evidence that general and PA-specific self-expansion decreased from baseline to follow-up (*M* = 5.04, *SD* = 13.53, *p* = 0.01 and *M* = 4.34, *SD* = 13.75, *p* = 0.02) and general and PA-specific self-expansion were similarly correlated at baseline and at follow-up (r = 0.74, *p *< 0.01 and r = 0.75, *p *< 0.01, respectively). Overall, participants were relatively active and averaged 8,781 steps per day (*SD* = 3,242), with daily step averages ranging from 4,059 to 19,376. Steps did not significantly change across the 4-week study (*p *= 0.51), which was expected as this was not an intervention, and participants were asked to maintain their usual activity levels. Baseline general self-expansion did not significantly predict average daily steps over the four weeks of the study (slope estimate = 20.68, SE = 30.12; *t*(47) = 0.69, *p* = 0.50), but baseline PA-specific self-expansion did significantly predict steps over the four weeks (slope estimate = 55.71, SE = 24.24; *t*(47) = 2.30, *p* = 0.03).

**Figure 1:**
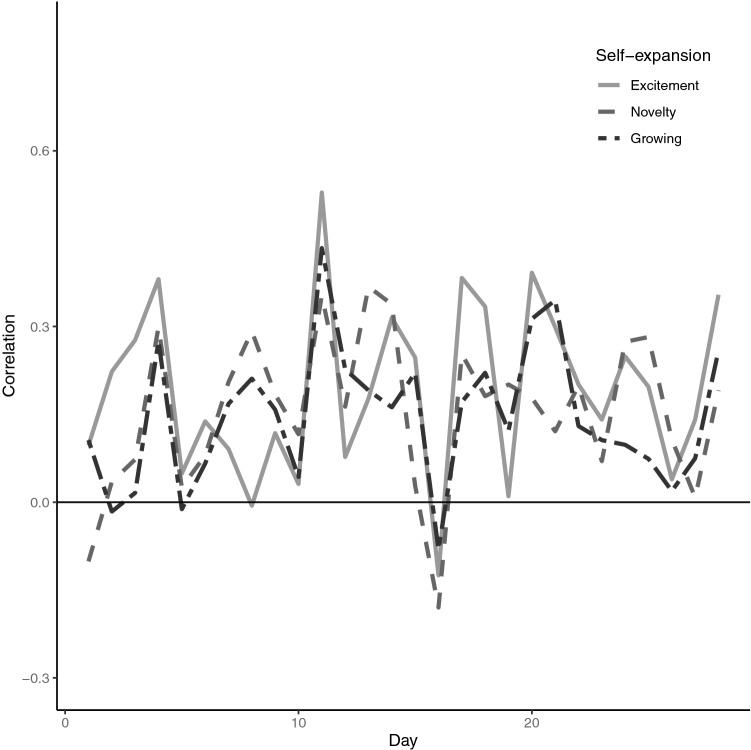
Daily correlations between daily steps and general self-expansion variables (solid light gray is self-expansion excitement, dashed gray is self-expansion novelty, and double-dash dark gray is self-expansion growing).

**Figure 2:**
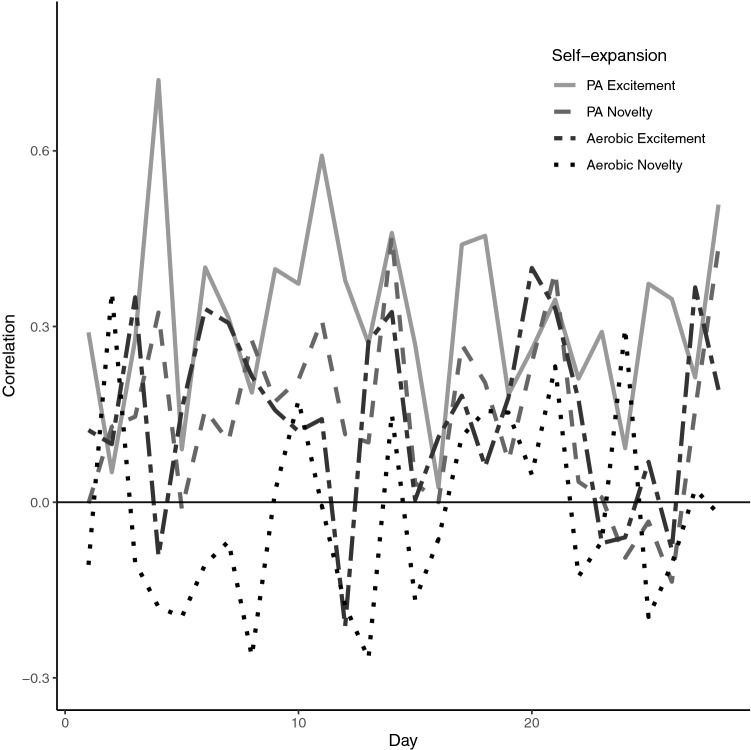
Daily correlations between daily steps and physical activity self-expansion variables (solid light gray is physical activity self-expansion excitement, dashed gray is physical activity self-expansion novelty, double-dash dark gray is aerobic excitement and dot-dash black is aerobic novelty).

Daily correlations between general self-expansion and steps ranged from − 0.18 (general novelty) to 0.53 (general excitement; Fig. 1) while correlations between PA-specific self-expansion and steps ranged from − 0.27 (PA-specific aerobic novelty) to 0.74 (PA-specific excitement; Fig. 2). Across the 4 weeks, steps taken was positively correlated with both general (all maximum likelihood *r* = 0.17) and PA-specific self-expansion (maximum likelihood *r* of 0.15 and 0.16). Additionally, daily steps was significantly positively correlated (maximum likelihood *r* of 0.38 and 0.50) with ratings of excitement and novelty about aerobic activity engaged in that day (see Table 1).

**Table 1:** Daily correlation between self-expansion measures and daily steps mean (standard deviation), maximum likelihood estimated correlation found using a mixed model, and test statistic and *p*-value for examining the association between Fitbit average daily steps and self-expansion using a mixed effects model. N = 49 for all analyses.

Domain	Daily question	Daily correlation mean (SD)	Maximum Likelihood Est. Correlation (r)	Interaction with Day *F* and *p*-value	Self-expansion main effect	Day main effect
General self-expansion	Did you feel excited?	0.19 (0.15)	0.17	*F*(27,1172) = 1.40, *p* = .084	*F*(1,1210) = 41.91, *p* < .001	*F*(27,1161) = 1.17, *p* = .252
	Did you feel that things were new?	0.16 (0.13)	0.17	* F*(27,1169) = 1.11, *p* = .316	*F*(1,1194) = 21.37, *p* < .001	*F*(27,1151) = 1.74, *p* = .010
	Did you feel that you were growing as a person?	0.15 (0.12)	0.17	*F*(27,1132) = 0.85, *p* = .691	*F*(1,1161) = 22.77, *p* < .001	*F*(27,1121) = 1.10, *p* = .330
PA-specific self-expansion	Did you feel excited about physical activity?	0.32 (0.16)	0.16	*F*(27,1112) = 1.45, *p* = .064	*F*(1,1194) = 98.72, *p* < .001	*F*(27,1102) = 1.60, *p* = .028
	Did you feel that physical activity offers something new to you?	0.15 (0.15)	0.15	*F*(27,1095) = 1.54, *p* = .038	*F*(1,1132) = 32.91, *p* < .001	*F*(27,1085) = 1.36, *p* = .104
	How exciting was this activity? (today’s aerobic activity)	0.15 (0.16)	0.38	*F*(27,435) = 0.88, *p* = .640	*F*(1,474) = 15.37, *p* < .001	*F*(27,432) = 0.60, *p* = .945
	How new was this activity? (today’s aerobic activity)	−0.02 (0.17)	0.50	*F*(27,444) = 0.53, *p* = .978	*F*(1,488) = 0.76, *p* = .385	*F*(27,431) = 1.29, *p* = .155

There was not sufficient evidence that the association between average number of steps and the general self-expansion measures differed depending on the day (interaction *p*-values between 0.084 and 0.691; Table 1). There was an association between steps and all general self-expansion measures (all *p*-values < 0.001). There was not sufficient evidence that the average number of steps for PA-specific excitement self-expansion measure differed depending on the day (interaction *F*(27,1112) = 1.45, *p* = 0.064). There was an association between steps and PA-specific excitement (*F*(1,1194) = 98.72, *p* < 0.001).There was evidence that the association between the average number of steps and PA-specific novelty self-expansion depended on time (interaction *F*(27,1095) = 1.54, *p* = 0.038). The association between average number of steps and PA-specific novelty on days 1 and 15 were negative (slope day 1 estimate = − 163.63, *p* = 0.830, slope day 15 estimate = − 659.6, *p* = 0.432) and the association between these two variables on all other days were positive (slope estimates range from 9.61 to 2543.49, *p* range from < 0.001 to 0.990). There was not sufficient evidence that the association between average number of steps and the aerobic activity measures depended on time (aerobic excitement *F*(27,435) = 0.88, *p* = 0.640; aerobic novelty *F*(27,444) = 0.53, *p* = 0.978). There was evidence of an association between number of steps and aerobic excitement (*F*(1,474) = 15.37, *p* < 0.001)) but not with aerobic novelty (*F*(1,488) = 0.76, *p* = 0.385).

## Discussion

This was the first study to examine self-expansion and a health behavior both at baseline and with daily observations. It was also the first study to examine both general and PA-specific self-expansion as well as the relationship between self-expansion and objectively-measured PA. The results of this study indicate a domain-specific baseline effect such that PA-specific self-expansion (but not general self-expansion) at baseline significantly predicted daily step. Both daily general self-expansion and PA-specific self-expansion were positively associated with average daily steps, with strong associations for PA-specific self-expansion items pertaining to aerobic activity. These findings suggest that both general and domain specific self-expansion could be fruitful for future research (e.g., for PA interventions, for studies on other health behaviors) and could be interesting to investigate for multiple behavior change.

While these results are encouraging, the study is not without limitations. This study examined self-expansion and daily steps in community adults and did not target particular groups (e.g., sedentary individuals). Future studies could investigate self-expansion and daily steps focusing on participants who are not currently meeting PA guidelines and who may benefit most from increases in PA. Since this was not an intervention study, we assessed self-expansion but did not attempt to manipulate self-expansion. Future research attempting to utilize self-expansion to increase PA should include the piloting of strategies to determine those that may be most effective. For example, researchers could ask target groups (e.g., sedentary individuals) to rate various forms of PA on novelty, excitement etc. Interventions could then focus on individuals engaging in the PA activities that they consider most self-expanding (compared to PA guidelines as usual and/or PA activities rated highly pleasant but not highly exciting or novel). To harness potential added benefits of domain specificity, individuals could identify self-expanding PA activities within specific categories (e.g., aerobic, weight-bearing, flexibility) and individuals could be asked to engage in each of these, perhaps in a variety of combinations over time to maintain novelty and excitement. This maintenance is important as habituation effects may lead to less self-expansion. For example, in this study both general self-expansion and PA self-expansion declined over the course of the four weeks. These declines were small in magnitude (e.g., 5 or 4 points on average out of 98 or 84), and while we cannot be sure of the cause of these declines, they may reflect the excitement and novelty of participating in the study and getting a new Fitbit wearing off over time.

The study utilized Fitbit which has been shown to provide valid steps data, however future research would benefit from using omni-directional accelerometers such as Actigraph. Further, this study focused on daily steps which cannot be generalized to PA. Future research can utilize Actigraph or other measures to more accurately assess multiple types of PA beyond just daily steps. As the present study was naturalistic and Fitbit devices are commonplace, no run-in period was employed, future studies (particularly those using less common measurement devices) may need to consider run-in periods. For this study, we recruited community adults in our region and our sample had restricted diversity, consisting of predominantly White non-Hispanic women. Future studies should include more diverse samples to help us understand which individual differences may be associated with stronger effects. We did not investigate individual’s employment type which could have provided interesting moderation information on the relationship between self-expansion and daily steps (e.g., if an individual’s job is particularly physically demanding and/or self-expanding). Finally, we did not assess for additional variables such as physical well-being in this project, future studies could examine these factors and their associations with self-expansion, PA, and the impact of these variables on the relationship between self-expansion and PA.

Because self-expansion is intrinsically motivating and does not require equipment or clinical staff, it may be a promising factor to investigate among under-resourced populations such as rural adults with health disparities and a strong need for cost-effective interventions. Since this study used a naturalistic design to detect the relationship between self-expansion and daily steps, the current data cannot speak to self-expansion causing an increase in daily steps. Self-expansion has been successfully manipulated in the past, although in a ‘relationships’ context rather than a health context, by assigning couples to engage in weekly highly self-expanding activities compared to pleasant control activities^11^. A next step in self-expansion and PA research would be to utilize a similar design to experimentally increase PA-specific self-expansion (assigning to highly self-expanding PA vs. pleasant PA) and examine acceptability, feasibility, and effectiveness of this intervention.

## Data Availability

A deidentified dataset has been deposited to the Open Science Framework: https://osf.io/y4r37/?view_only=0c5c4a7015354a169ea0cd4534a3847f.
